# Integrated analysis of microRNA regulatory network in nasopharyngeal carcinoma with deep sequencing

**DOI:** 10.1186/s13046-016-0292-4

**Published:** 2016-01-22

**Authors:** Fan Wang, Juan Lu, Xiaohong Peng, Jie Wang, Xiong Liu, Xiaomei Chen, Yiqi Jiang, Xiangping Li, Bao Zhang

**Affiliations:** Department of Otolaryngology, Head and Neck Surgery, Nanfang Hospital, Southern Medical University, Guangzhou, 510515 China; School of Public Health and Tropical Medicine, Southern Medical University, Guangzhou, 510515 China; Department of Guangdong No.2 District, BGI Genomics Co., Ltd, Shenzhen, 518083 China

**Keywords:** Nasopharyngeal Carcinoma, microRNA, Deep Sequencing, Regulatory network, miR-34c-5p

## Abstract

**Background:**

MicroRNAs (miRNAs) have been shown to play a critical role in the development and progression of nasopharyngeal carcinoma (NPC). Although accumulating studies have been performed on the molecular mechanisms of NPC, the miRNA regulatory networks in cancer progression remain largely unknown. Laser capture microdissection (LCM) and deep sequencing are powerful tools that can help us to detect the integrated view of miRNA-target network.

**Methods:**

Illumina Hiseq2000 deep sequencing was used to screen differentially expressed miRNAs in laser-microdessected biopsies between 12 NPC and 8 chronic nasopharyngitis patients. The result was validated by real-time PCR on 201 NPC and 25 chronic nasopharyngitis patients. The potential candidate target genes of the miRNAs were predicted using published target prediction softwares (RNAhybrid, TargetScan, Miranda, PITA), and the overlay part was analyzed in Gene Ontology (GO) and Kyoto Encyclopedia of Genes and Genomes (KEGG) biological process. The miRNA regulatory network analysis was performed using the Ingenuity Pathway Analysis (IPA) software.

**Results:**

Eight differentially expressed miRNAs were identified between NPC and chronic nasopharyngitis patients by deep sequencing. Further qRT-PCR assays confirmed 3 down-regulated miRNAs (miR-34c-5p, miR-375 and miR-449c-5p), 4 up-regulated miRNAs (miR-205-5p, miR-92a-3p, miR-193b-3p and miR-27a-5p). Additionally, the low level of miR-34c-5p (miR-34c) was significantly correlated with advanced TNM stage. GO and KEGG enrichment analyses showed that 914 target genes were involved in cell cycle, cytokine secretion and tumor immunology, and so on. IPA revealed that cancer was the top disease associated with those dysregulated miRNAs, and the genes regulated by miR-34c were in the center of miRNA-mRNA regulatory network, including TP53, CCND1, CDK6, MET and BCL2, and the PI3K/AKT/ mTOR signaling was regarded as a significant function pathway in this network.

**Conclusion:**

Our study presents the current knowledge of miRNA regulatory network in NPC with combination of bioinformatics analysis and literature research. The hypothesis of miR-34c regulatory pathway may be beneficial in guiding further studies on the molecular mechanism of NPC tumorigenesis.

**Electronic supplementary material:**

The online version of this article (doi:10.1186/s13046-016-0292-4) contains supplementary material, which is available to authorized users.

## Background

Nasopharyngeal carcinoma (NPC) is a highly invasive and metastatic cancer that is widely prevalent in southern China [1, 2]. During tumorigenesis and progression, multiple genetic and epigenetic abnormalities synergistically disrupt normal cell function, especially playing an important role in NPC pathogenesis [3, 4]. In recent decades, microRNAs (miRNAs) have been increasingly recognized as important genetic regulators in the mammalian system [5]. Dysregulation of miRNA expression in NPC can regulate tumor cell growth, differentiation and apoptosis.

MiRNAs are endogenous, small (18–25 nt), non-protein-coding RNA molecules which modulate many physiological and pathological processes through down-regulating target genes [6]. Previous studies mostly focused on single miRNA, such as miR-138, a potential tumor suppressor by targeting CCND1 [7]. But microRNA expression profiles have been demonstrated to be unique for a better understanding of different stages of tumor progression and metastasis. Hence, it is necessary to use high-tech tools to get a comprehensive miRNA analysis of NPC etiology.

Thus far, several high-throughput techniques have been applied to identify deregulated miRNAs in NPC. Most studies to date have applied microarray technique on nasopharyngeal carcinoma and non-cancer nasopharyngitis tissues, and some of them could identify the miRNAs as a prognostic factor or recurrent marker from the distinctive miRNA expression profile [8–10]. But the results failed to show good interplatform concordance. Both Sengupta et al. and Luo et al. recruited microarray on laser-microdissected tissues which including NPC and normal surrounding epithelial cells, only three miRNAs (miR-34c-5p, miR-29c and miR-34b-5p) were shared in the list of abnormal expressions by comparing Sengupta’s data and Luo’s data [11, 12]. The inconsistency may be explained by heterogeneity of samples and use of different expression measuring platforms. To get the better data reproducibility, we need some more robust tools to reduce the disturbance of heterogeneity.

The advent of the deep sequencing provides a rapid and high throughput tool that can be used to explore the large miRNA pool, and possesses obvious advantages for the identification of miRNA sequence variations and the discovery of novel miRNAs [13]. Plieskatt et al. compared miRNA expression in FFPE ( formalin fixed paraffin- embedded tissue) from NPC cases and controls using both microarray and RNA-Seq technologies, showed that RNA-Seq could additionally indicate unknown miRNAs associated with NPC [14]. Deep sequencing technology was considered as a more powerful and accurate tool in expression profile study, which would allow us to generate a comprehensive insight into the networks constructed by many more deregulated miRNAs not described in previous studies, just like the study of Chang et al., which delivered a clear picture of the global miRNA regulatory characteristics in triple-negative breast cancer by deep sequencing [15].

To better characterise the specific signature of NPC cells, we expand our observations by applying laser capture microdissection (LCM) on NPC and chronic nasopharyngitis samples to compare the miRNA signatures between them. LCM can separate the truly transformed cancerous cells from those other cell types commonly present in a tumor tissue, such as immune cells, connective tissue and new vasculature [16–18]. Wang et al. demonstrated the feasibility and potential power of discovering miRNA biomarkers in colorectal tumor tissue using the combination of LCM with miRNA microarrays [19], and with the combination of LCM and microarray, Sengupta et al. identified miR-29c was important in regulating genes involved in metastasis of NPC [11].

Herein, we aim to examine the miRNA expression profile in clinical tissue samples of NPC patients, and get better understanding of the miRNA regulatory network. After bioinformatics analysis and literature review, we identify miR-34c as an important partner in NPC tumorigenesis, and propose that miR-34c-target genes network (including CCND1, TP53, BCL2, CDK6, MET) should be critical in mediating NPC cellular proliferation, migration and invasion.

## Methods

### Patient samples and laser capture microdissection

A total of 213 NPC patients and 33 chronic nasopharyngitis patients were recruited prospectively from Nanfang Hospital (Southern Medical University, Guangzhou, China). None of the patients had received anticancer treatments before undergoing biopsy. Fresh tissues were snap frozen in liquid nitrogen and stored at −160 °C. All samples were pathologically confirmed. The patients were informed about the sample collection and had signed informed consent forms. TNM classification was according to the definitions of the seventh edition of the UICC-American Joint Committee on Cancer staging criteria. 12 NPC biopsy specimens and 8 non-cancer nasopharyngitis biopsy samples were chose for laser capture microdissection (LCM). The controls were carefully selected to match the gender and age distribution of NPC patients. Because the sample of stage I failed to pass the quality control, we chose 2 cases of stage II, and 5 cases of each stage III IV (Table 1). Specimens were first frozen-sectioned by using a LEICA CM 1900 cryomicrotome. After hematoxylin and eosin (H&E) staining, LCM was performed on a MMI Cellcut Microdissection Instrument (Molecular Machines & Industries, Swiss). Phase contrast images were acquired using OlympusIX71 microscope. The research protocols were approved by the Ethics Committee of Nanfang Hospital and registered in Clinical.trials.gov (ID: NCT01171235).

**Table 1:** Patient characteristics in laser microdissected samples

	NPC	Controls
Sex		
Male	9	6
Female	3	2
Age(Mean)	49	44
Stage		
II	2	
III	5	
IV	5	

### Small RNA deep sequencing

Total RNA, containing miRNA, was extracted using Trizol Reagent (Invitrogen, CA) from laser-microdessected samples, and passed the RNA quality control for sequencing. The quality and integrity of total RNA was assessed with Agilent 2100 Bioanalyzer (Agilent Technologies, USA). To achieve optimal tissue miRNA profiles, we carried out high-throughput next-generation sequencing (Illumina, BGI, Shenzhen) of 12 NPC samples and 8 chronic nasopharyngitis samples by following the manufacturer’s recommended protocols. We screened the high quality clean read sequences by the alignment to NCBI GenBank data and miRBase 21.0 for the further analysis. The raw data have been submitted to NCBI under BioProject accession No.PRJNA 289899.

### Differential miRNA expression analysis

To identify miRNAs differentially expressed between NPC patients and controls, we applied transcripts per million (TPM) to normalize the expression of miRNA in two groups (NPC and controls). Then we calculated fold change (FC) and P-value via T-test, and corrected P-value into false discovery rate (FDR) using the Benjamin and Hochberg method [20]. FDR ≤ 0.05 and | log2FC | ≥ 2 were set as the cut-offs to screen out differentially expressed miRNAs.

### Quantitative reverse transcription–PCR analysis of miRNA expression levels

Quantitative reverse transcription-PCR was used to validate the sequencing results. Total RNA from 201 NPC and 25 chronic nasopharyngitis biopsy specimens was extracted using Trizol Reagent (Invitrogen, CA). Reverse transcription of the total RNA was performed using All-in-One First-Strand cDNA Synthesis kit (GeneCopoeia Inc., USA) according to the manufacturer’s protocol. Real-time PCR was performed by using All-in-OneTM qPCR Mix (Applied GeneCopoeia Inc., USA) on Roche Lightcycler 480 System. U6 snRNA was used as miRNA endogenous control respectively. All samples were normalized to internal control and fold changes were calculated through relative quantification [21].

### Bioinformatics analysis

Four softwares (RNAhybrid, TargetScan, Miranda, PITA) were used for target gene prediction and only the genes identified by all four approaches were selected out [22–25]. And we chose the overlapped genes targeted by oncogenic miRNAs for further study, as well as the tumor suppressor miRNAs. To understand the functions of predicted target genes, Gene ontology (GO) and Kyoto encyclopedia of genes and genomes (KEGG) enrichment analysis were performed with package GO stats (http://www.geneontology.org/) of *P* value <0.05 was set as the cut-off to select out significantly enriched terms [26]. Then the miRNA regulatory network analysis was performed using the IPA software (http://www.ingenuity.com). The created genetic networks describe functional relationships among miRNAs and genes based on known associations in the databases [27]. Networks related were ranked according to their biological relevance to the gene list provided.

### Oligonucleotide transfection

Human nasopharyngeal carcinoma cell line CNE-2 was cultured in RPMI-1640 (HyClone, Thermo scientific Inc, China) supplemented with 10 % fetal bovine serum (FBS, Gibco, Grand Island, NY, USA). CNE-2 was transfected with miR-34c mimic or miR-Ctrl (20 nM; Ribobio, Guangzhou, China) using lipofectamine 2000 reagent (Invitrogen, Carlsbad, CA, USA). The cells were harvested for assays 48 h after transfection.

### Western blot

Proteins lysates extracted from cells were separated by 6 and 10 % SDS-PAGE, and electrophoretically transferred to PVDF (polyvinylidene difluoride) membrane (Millipore). Then the membranes were incubated with the following antibodies: Met, Bcl-2, CDK6 (1:1000; Cell Signaling Technology, Beverly, MA, USA) and CCND1(1:500; ABclonal, College Park, MD, USA), and followed by HRP (horseradish peroxidase)-labeled goat anti-rabbit IgG (1:5000; Liankebio, Hanzhou, China) as secondary antibody. The blots were visualized using the electrochemiluminescence detection system. β-actin (1:1000; Cell Signaling Technology, Beverly, MA, USA) was used as a protein loading control.

### Statistical analysis

Statistical analyses were conducted using spss19.0 software. The data are shown as the mean ± SEM unless otherwise noted. Two-tailed Student’s *t* test was used for identify differentially expressed miRNAs between NPC and controls. One-way ANOVA was used to show significant associations between the miR-34c-5p level and clinicopathological parameters. *P* values of <0.05 were considered statistically significant.

## Results

### Differentially expressed miRNAs between NPC and controls

**Fig. 1:**
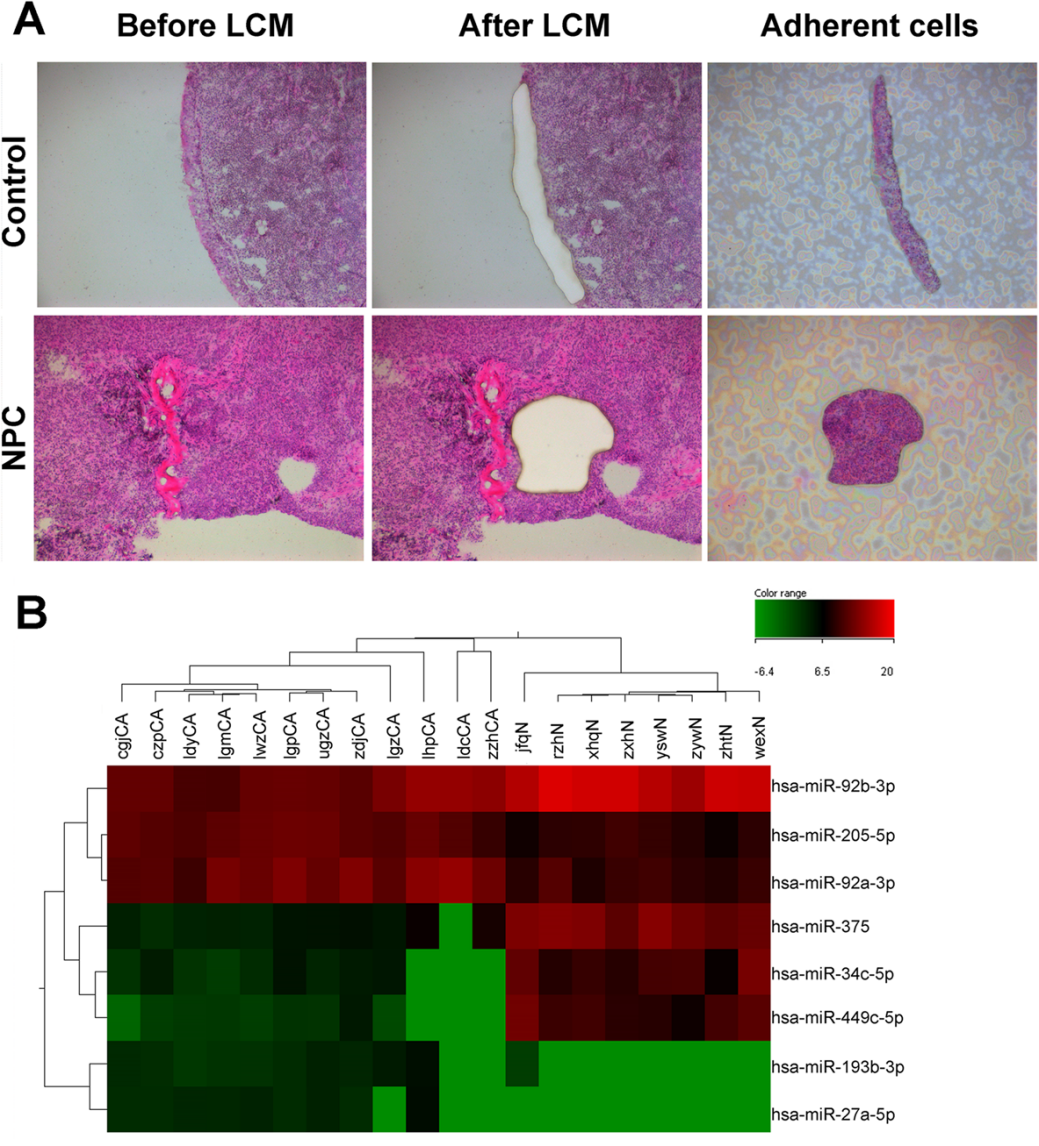
MiRNA differential expression. **a** Laser capture microdessection of H&E-stained slides (×10). **b** Heatmap of normalized miRNA reads that are differentially expressed between NPC and controls (sequencing-read count ratio ≥ 2.0, *P* < 0.05). Samples are well divided into control and NPC patient groups

In order to isolate tumor cells from non-tumor cells, LCM was performed on NPC and chronic nasopharyngitis specimens (Fig. 1a). After normalization and T-test on the sequencing data, 8 differentially expressed miRNAs were screened, 4 up-regulated (miR-205-5p, miR-92a-3p, miR-193b-3p and miR-27a-5p) and 4 down-regulated (miR-34c-5p, miR-375, miR-92b-3p and miR-449c-5p) (Table 2). Cluster analysis demonstrated that these miRNAs could well distinguish the NPC samples from controls (Fig. 1b). But the novel miRNAs mostly had individual specificity, were not suitable for further analysis.

**Table 2:** Top 8 differentially expressed miRNAs

miRNA	Average TPM in Control	Average TPM in NPC	Log2(FC)	FDR
hsa-miR-27a-5p	0.03	79.7867	353.84	0.017
hsa-miR-193b-3p	1.403	73.4603	257.719	0.03
hsa-miR-92a-3p	4795.317	24268.259	4.5575	7.14E-3
hsa-miR-205-5p	3398.737	13008.145	3.9103	7.68E-4
hsa-miR-92b-3p	240483.065	25576.241	−10.7984	3.36E-4
hsa-miR-375	25725.996	405.155	- 226.8843	8.76E-3
hsa-miR-34c-5p	8338.523	79.437	- 565.0915	8.25E-3
hsa-miR-449c-5p	7473.709	24.586	−1968.9108	5.82E-4

*Abbreviations*: *TPM* transcripts per million, *Control* chronic nasopharyngitis sample, *NPC* nasopharyngeal carcinoma, *FC* fold change

### Validation of expression of miRNAs

**Fig. 2:**
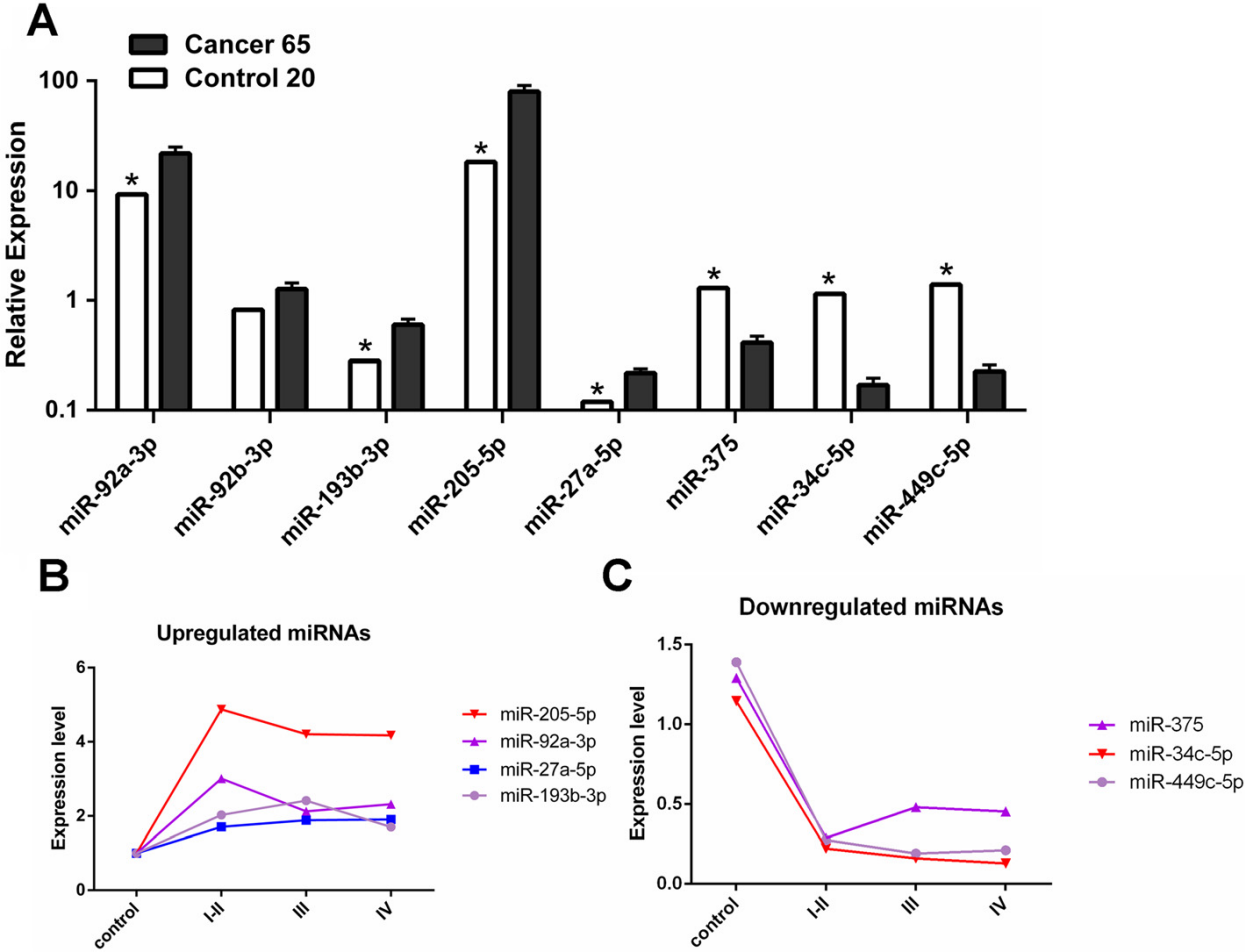
Validation of deregulated miRNAs by qRT-PCR. **a** An independent validation cohort included 65 NPC patients and 20 healthy control subjects. **b** Dynamic expression of supregulated miRNA. **c** Dynamic expressions of downregulated miRNA. MicroRNA abundance was normalised to U6 RNA. Statistical analysis was performed using the t-tests. *P < 0.01

To confirm the deep sequencing results, we used qRT-PCR to assess the expression of 8 miRNAs with an independent cohort (including 65 NPC patients and 20 chronic nasopharyngitis patients). The relative level of each miRNA was shown in Fig. 2a. Expression of 7 miRNAs measured by qRT-PCR was dramatically different between NPC and chronic nasopharyngitis tissues and significantly correlated with their sequencing data. Because miR-92b-3p was not reliably measured by qRT-PCR in the tissue specimens, it was excluded from further analysis. The dynamic expression levels of miRNAs were revealed that the patterns were classified into 2 groups, and the varying tendencies between control and NPC were consistent with the sequencing result (Fig. 2b, c). Moreover, the expression level of miR-34c decreased with the ascent of clinical stage, which implicated that miR-34c might be more important in NPC progression.

### Prediction and function analysis of target genes of miRNAs

Functional analysis was conducted on genes predicted as targets of 7 differentially expressed miRNAs by taking the overlay part of 4 software results (RNAhybrid, TargetScan, Miranda, PITA). 666 genes targeted by downregulated miRNAs and 248 genes targeted by upregulated miRNAs were acquired after screening (Additional file 1: Table S1, S2). The selected genes were analyzed by the Gene ontology (GO) enrichment and Kyoto encyclopedia of genes and genomes (KEGG) pathway analysis.

The enrichment analysis of GO categories included biological process (BP), molecular function (MF), and cellular component (CC) three parts. The highest enrichment were the terms like “Interleukin-1 secretion”, “Mast cell activation involved in immune response”, “Fibroblast growth factor binding”, “Lipopolysaccharide binding” and “Cortical actin cytoskeleton” (Table 3). These results showed the function of target genes mostly focused on cell growth, secretion, migration and immune system process.

**Table 3:** GO enrichment analysis of the target genes

	ID	Term	Enrichment	FDR
Cellular component	GO:0030133	Transport vesicle	9.10473	2.50E-07
	GO:0019898	Extrinsic to membrane	7.53782	0.0001
	GO:0030864	Cortical actin cytoskeleton	21.17768	0.00036
	GO:0048770	Pigment granule	5.527732	0.00066
Biological process	GO:0048278	Vesicle docking	21.30266	1.95E-08
	GO:0051047	Positive regulation of secretion	15.36585	6.06E-07
	GO:0050701	Interleukin-1 secretion	66.95122	1.86E-06
	GO:0002767	Immune response-inhibiting cell surface receptor signaling pathway	29.28	5.40E-06
	GO:0002279	Mast cell activation involved in immune response	52.07317	1.09E-05
	GO:0032512	Regulation of protein phosphatase type 2B activity	27.328	0.00456
	GO:0032957	Inositol trisphosphate metabolic process	28.84053	0.0057
Molecular function	GO:0035091	Phosphatidylinositol binding	11.07261	5.92E-09
	GO:0008289	Lipid binding	3.68819	1.03E-06
	GO:0034185	Apolipoprotein binding	18.24711	8.53E-06
	GO:0017134	Fibroblast growth factor binding	39.21549	1.60E-05
	GO:0004520	Endodeoxyribonuclease activity	13.72542	0.00011
	GO:0001530	Lipopolysaccharide binding	34.22443	0.00063
	GO:0015377	Cation:chloride symporter activity	10.70131	0.00387
	GO:0016802	Trialkylsulfonium hydrolase activity	19.36428	0.00752
	GO:0015140	Malate transmembrane transporter activity	33.88748	0.00785
	GO:0032396	Inhibitory MHC class I receptor activity	33.88748	0.00785

The result of KEGG pathway analysis was enumerated in Table 4. Chemokine signaling pathway, Cytokine-cytokine receptor interaction and Cell cycle, Natural killer cell mediated cytotoxicity, etc. were remarkable pathways which could further confirm the target genes’ function in tumor cell proliferation, secretion and tumor immunology. So the differentially expressed miRNAs might be involved in the development of NPC by targeting these genes.

**Table 4:** The KEGG pathway analysis of the target genes

Term	ID	Count	Percentage	*P*-value
Up-regulated miRNAs target gene KEGG-PATHWAY				
Inositol phosphate metabolism	ko00562	11	4.51	3.94E-6
Phopphatidylinositol signaling system	ko04070	11	4.51	1.84E-4
Chemokine signaling pathway	ko04062	12	4.92	4.58E-3
Down-regulated miRNAs target gene KEGG-PATHWAY				
Natural killer cell mediated cytotoxicity	ko04650	34	4.96	4.37E-9
Antigen processing and presentation	ko04612	21	3.07	1.46E-8
Galactose metabolism	ko00052	10	1.46	7.58E-5
Cell cycle	ko04110	17	2.48	5.07E-3
Cytokine-cytokine receptor interaction	ko04060	22	3.21	0.01523

### Regulatory networks for differentially expressed miRNAs

**Fig. 3:**
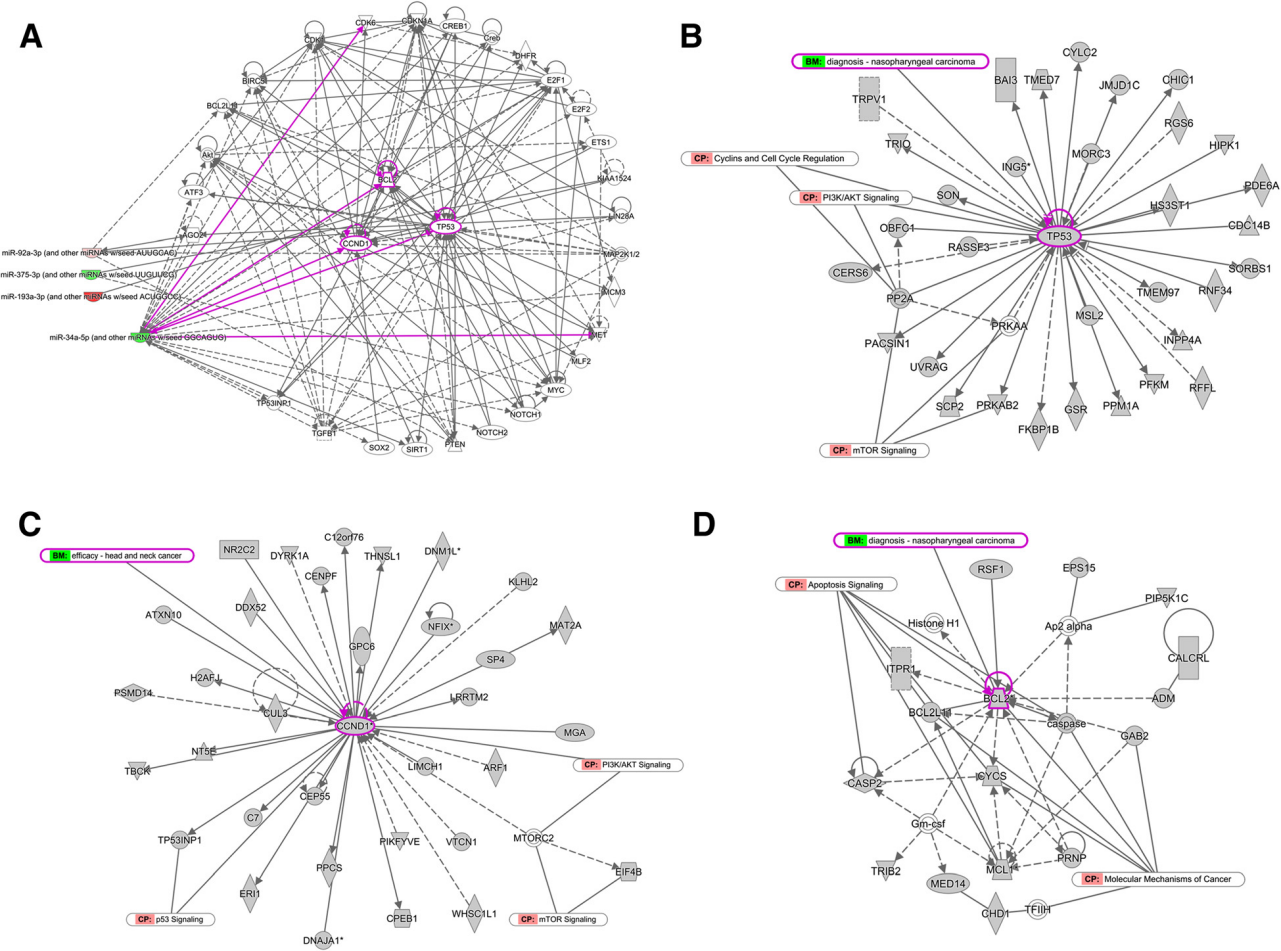
MiRNAs regulatory networks by IPA. **a** The miRNA-mRNA interaction network. miR-34 family targeted most genes and the relationship between it and 5 highlighted genes (CCND1, TP53, BCL2, MET, CDK6) was experimentally confirmed. **b** TP53-centered network showed TP53 could be treated as diagnostic marker of NPC and was involved in cell cycle regulation and mTOR signaling. **c** CCND1- centered network indicated CCND1 was involved in both p53 signaling and PI3K/AKT/mTOR signaling. **d** BCL2-centered network showed BCL2 acted significantly in molecular mechanisms of cancer and functioned through apotosis signaling

The biological function analysis of target genes from Ingenuity pathway analysis (IPA) database showed that cancer was the top functional category which was significantly related to the ectopic miRNAs, along with embryonic and organismal development, which also could be associated with cancer (Table 5). The filtering used in IPA allowed us to connect 6 miRNAs with target genes. In the regulation network of miRNA, miR-34 family (including miR-34c-5p, miR-449c-5p) targeted the most genes, and 5 directions of miR-34c come from experimental observations were highlighted (Fig. 3a). That indicated the relationships between miR-34c and the 5 genes (TP53, CCND1, BCL2, CDK6, MET) in cancer have already been confirmed. The binding sites of miR-34c on these 5 genes could be found through TargetScan software (Additional file 2: Figure S1). CCND1, TP53 and BCL2 were clearly in the center of network, so their related networks were picked out for pathway construction. TP53 has been largely confirmed as a tumor suppressor gene that mediated cell cycle regulation with its downstream factors and could be treated as diagnostic marker of NPC (Fig. 3b). And CCND1 and TP53 were both involved in PI3K/AKT/mTOR signaling that modulated tumor cells growth and proliferation (Fig. 3b, c). Sometimes they even induced apoptosis or autophagy. Base on the IPA data, BCL2 could also be predicted to be the diagnostic marker of NPC as TP53, while it acted significantly in molecular mechanisms of cancer and functioned through apoptosis signaling (Fig. 3d).

**Table 5:** IPA analysis of target genes according to 7 dysregulated miRNAs

Top Networks(score)	Top Diseases and Disorders
Cancer, Embryonic Development, Organismal Development (27)	Cancer
Cardiovascular Disease, Developmental Disorder, Pulmonary Hypertension (27)	Organismal Injury and Abnormalities
Cell Morphology, Cellular Assembly and Organization, Embryonic Development (27)	Gastrointestinal Disease
Cancer, Organismal Injury and Abnormalities, Cellular Growth and Proliferation (27)	Hepatic System Disease
Cancer, Dermatological Diseases and Conditions, Organismal Injury and Abnormalities (25)	Reproductive System Disease

### Comparison of miR-34c levels during NPC progression

**Fig. 4:**
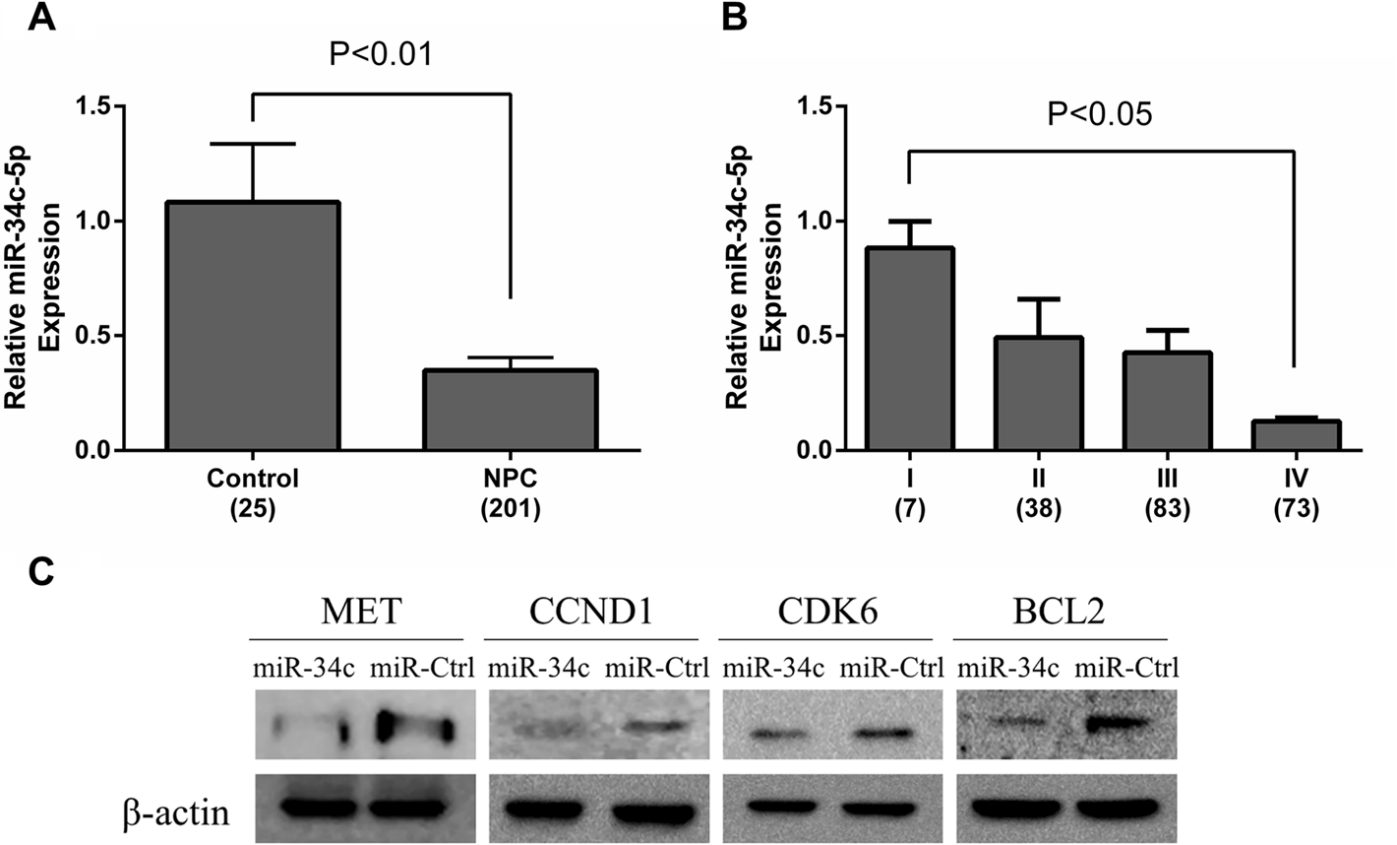
MiR-34c levels during NPC progression and target genes validation. **a** The expression level of miR-34c-5p in human NPC specimens compared with control biopsy samples. **b** miR-34c-5p expression was higher in stage I, whereas stages II-IV had lower levels. **c** The protein expression levels of MET, CCND1, CDK6 and BCL2 in miR-34c mimic transfected CNE-2 cells were lower than the controls. Western blot was independently repeated at least three times. MicroRNA abundance was normalised to U6 RNA. Statistical analysis was performed using the t-tests (**a**, **c**) and the one-way ANOVA (**b**)

As the previous results showed miR-34c might be more valuable than others, miR-34c levels in more tissue samples using qRT–PCR were further validated. A total of 201 NPC tissue samples were used to validate miR-34c via qRT-PCR, it was significantly reduced in NPC patients as compared with controls (*P* < 0.01, Fig. 4a), and showed the significant correlation with clinical stages respectively (*P* < 0.05, Fig. 4b). All these data suggested that low concentration of miR-34c was associated with higher tumour stage.

### MiR-34c inhibited MET, CCND1, CDK6, BCL2 expression in NPC cells

To further characterize whether these target genes (MET, CCND1, CDK6, BCL2) respond to miR-34c in NPC cells, CNE-2 cells were transfected with miR-34c mimic or miR-Ctrl. Western blot was performed to detect the expression level of MET, CCND1, CDK6 and BCL2. As shown in Fig. 4c, the overexpression of miR-34c with miR-34c mimics led to a notable decrease in these four genes protein levels compared with the negative control in CNE-2 cells. These results indicated that MET, CCND1, CDK6 and BCL2 were actually targeted by miR-34c in NPC cells.

## Discussion

We analyzed the miRNA expression profile from 12 NPC and 8 chronic nasopharyngitis tissues after LCM with the RNA-seq technology in this study. Deregulated miRNAs were screened from the statistical analyses and a panel of 7 significantly differentially expressed miRNAs (miR-34c-5p, miR-375, miR-449c-5p, miR-205-5p, miR-92a-3p, miR-193b-3p and miR-27a-5p) was found to be an effective regulatory factor between NPC and controls. And our result was also consistent with many studies of other tumors, including miR-34c and miR-375, which were both downregulated in colorectal carcinoma [28, 29], and miR-449c was showed to inhibit gastric carcinoma growth [30]. On the contrary, miR-205, miR-92a, miR-193b and miR-27a-5p were reported to be upregulated in other kinds of cancers, including lung cancer, cervical cancer, glioma and renal cell carcinoma [31–34].

According to our expression profile of miRNA, there is no study about the deregulated expression of miR-27a-5p, miR-193-3p, miR-449c-5p or miR-92a-3p in NPC yet. Mir-375 expression was significantly reduced in NPC and inhibited tumor growth by targeting MTDH [35]. The results of Tang et al. implicated miR-205-5p may be a novel NPC candidate biomarker, which was showed to determine the radioresistance of NPC through the miR-205-PTEN-Akt pathway [36]. The reduced expression of miR-34c frequently occurred in many studies of NPC, which was believed to play important role in NPC. The recent study of Li et al. proved that miR-34c suppressed NPC cell viability, colony formation by targeting MET [37]. Our study also showed the miR-34c level in NPC tissues got a decreasing trend during NPC progression via qRT-PCR.

Nevertheless, it has been known that miRNAs always functioned not only through one single gene, but a huge network which covered multiple genes. For a deeper understanding of the functional genomic alterations induced by deregulated miRNAs, target genes were predicted by multiple softwares included RNAhybrid, TargetScan, Miranda and PITA, and the bioinformatics analysis of the functional category and integrated network were conducted, including GO, KEGG enrichment analysis and IPA. The GO enrichment analysis found that the target genes of these dysregulated miRNAs were associated with cell growth, protein binding and secretion, which were all involved in tumorigenesis, KEGG analysis also obtained similar results (Table 3, Table 4). Interestingly, the “Natural killer cell mediated cytotoxicity” was the top term in KEGG analysis and the genes involved in this process were all targeted by miR-34c, which meant tumor immunology should not be ignored in NPC pathogenesis. Recent studies showed miR-34c could affect NK cell’s activation in melanoma [38], indicating an exciting future in the field of NK cell-based cancer immunotherapy [39].

MiRNAs regulatory network from IPA data showed the relationships between 5 target genes (TP53, CCND1, BCL2, CDK6, MET) and miR-34c in various cancers were experimental confirmed and located in the center of network (Fig. 3a). TP53, CCND1 and BCL2 were demonstrated to modulate tumor cells growth and proliferation via PI3K/AKT/mTOR signaling. These results indicated the downregulation of miR-34c might play an important role in NPC.

TP53 is predicted to be a diagnostic marker of NPC in IPA, which functions as a tumor suppressor protein that can induce cell cycle arrest and apoptosis. It was deemed to positively play a synergetic role in suppressing NPC tumorigenesis with miR-34 family not like miR-125a/b or miR-BHRF-1 which directly downregulated TP53 [40–42]. This further demonstrates that in the pathogenesis of NPC, even a single gene can be regulated by many factors, the exploration of miRNA regulatory network is especially vital in miRNA integrated analysis of NPC.

CCND1, CDK6 and BCL2 are all cell cycle regulators. CCND1 usually functions as a regulatory subunit of CDK6, whose activity is required for cell cycle G1/S transition and thought to be an oncogene in various tumors including NPC. The expression level of CCND1 was related to radiosensitivity in NPC treatment [43]. Previous studies showed EBV could encoded LMP1 to enhance the promoter activity of CCND1 in NPC, and miR-144 suppressed the expression of PTEN to increase the expression of CCND1 to promote tumor migration and invasion [44, 45]. Our results indicated that CCND1 might be a target gene of miR-34c in NPC, which should be further confirmed in future. As one target of CCND1, CDK6 is important for cell cycle G1 phase progression and G1/S transition, while miR-26a can suppress CDK6 expression through EZH2 to inhibit tumorigenesis of NPC [46]. BCL2 acted as the anti-apoptotic protein by increasing the rate of cell division and arresting cells in the G0/G1 checkpoint of the cell cycle, and it could also be treated as a diagnosis marker as TP53 [47]. Mir-21 could target BCL2 directly to suppress NPC cells proliferation and migration, while PDCD4 regulated its expression level via modulating miR-184 [48, 49].

The mechanism of MET in many tumors has been well explored in various research [50, 51]. In NPC, miR-34c had been clearly proved to target MET [37], and it was activated by hepatocyte growth factor (HGF), then modulated cellular proliferation, migration, invasion via inhibition of EGR-1 expression [52, 53]. Furthermore, PHA-665752, MET inhibitor, could furthermore effectively affect the radiosensitivity of NPC and suppress NPC cells proliferation [54]. Hence, Li et al. showed MET protein overexpression and gene amplification were independent prognostic factors and might be treated as therapeutic biomarkers in NPC [55].

**Fig. 5:**
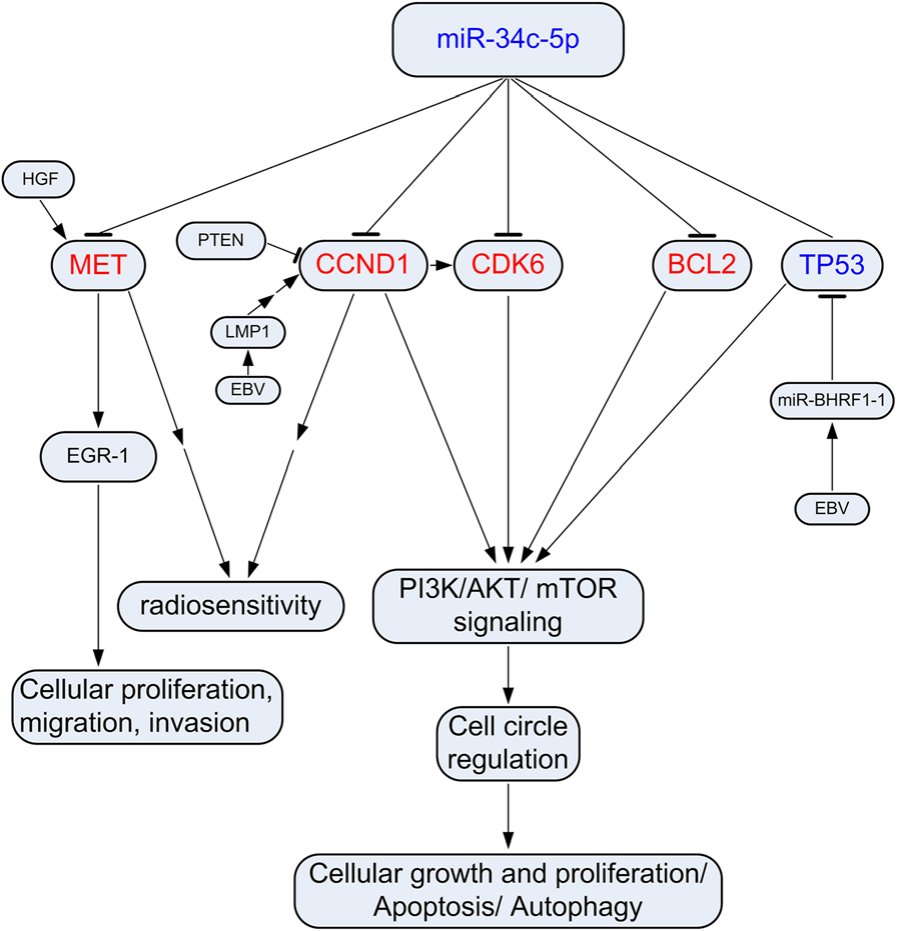
MiR-34c regulatory network. Blue fonts represent tumor supressor factors while red fonts represent oncogenic factors. The interaction between miRNAs and mRNAs demonstrate miR-34c-5p might regulates NPC cellular growth and migration, apoptosis via these genes and functions through PI3K/AKT/mTOR signaling. → Direct Stimulatory Modification, → → Multistep Stimulatory Modification, −Direct Inhibitory Modification, −Tentative Associated Modification

Above all mentioned, the PI3K/AKT/mTOR signaling was considered as the function pathway of deregulated miRNAs to mediate cellular growth and proliferation, apoptosis and autophagy. CCND1, CKD6, BCL2 and TP53 worked together on cell circle regulation, while MET was also significant in NPC cellular proliferation, migration and invasion, and might work through other pathways. MiR-34c was thought to be the key factor in this network. And our qRT-PCR results further confirmed the expression level of miR-34c decreased with the ascent of tumor stage (Fig. 4b). So we hypothesized miR-34c regulated NPC development through the network constructed by these factors and inferred their mechanism in NPC respectively in this model (Fig. 5).

## Conclusions

In conclusion, our study disclosed a global profiling of differentially expressed miRNAs that was closely related with NPC carcinogenesis and progression. For a better understanding of the miRNA regulation on genome-wide repression and activation, we use integrated approaches including GO, KEGG and IPA. In the miRNA regulatory network, miR-34c and its target genes including TP53, CCND1, BCL2, CDK6 and MET are shown to play important roles in NPC, and the PI3K/AKT/mTOR signaling is regarded as a significant function pathway mediated by miR-34c. Among these differentially expressed miRNAs, miR-34c expression decreases during NPC development, indicating it as a potential biomarker monitoring NPC progression. Thus, miR-34c may be the key modulator in the process of tumorigenesis and treated as the biomarker of NPC in future research.

## Additional files

Additional file 1: Table S1. The genes targeted by downregulated miRNAs. **Table S2**. The genes targeted by upregulated miRNAs. (XLS 113 kb) Additional file 2
**Figure S1.** MiR-34c-5p have binding sites on the 3’-UTR of target genes. A. Complementary binding sites of miR-34c-5p on the 3’-UTR of CCND1. B. Complementary binding sites of miR-34c-5p on the 3’-UTR of BCL2. C. Complementary binding sites of miR-34c-5p on the 3’-UTR of CDK6. D. Complementary binding sites of miR-34c-5p on the 3’-UTR of MET. The vertical lines indicate the complementary base pairs (which have also been highlighted). (DOC 5879 kb) Additional file 3: Figure S2. The distribution plot for differentially expressed miRNAs. (TIF 103 kb) Additional file 4: Table S3. miRNA expression matrix. (XLSX 119 kb) Additional file 5: Table S4. T-test results of differentially expressed miRNAs. (XLSX 10 kb)

## Abbreviations

NPC: nasopharyngeal carcinoma
miRNA: microRNA
GO: gene ontology
KEGG: kyoto encyclopedia of genes and genomes
IPA: ingenuity pathway analysis
EBV: epstein-barr virus
LCM: laser capture microdissection
H&E: hematoxylin and eosin staining
TPM: transcripts per million
FC: fold change
FDR: false discovery rate

## References

[1] Yoshizaki T, Ito M, Murono S, Wakisaka N, Kondo S, Endo K (2012). Current understanding and management of nasopharyngeal carcinoma. Auris Nasus Larynx.

[2] Henderson BE (1974). Nasopharyngeal carcinoma: present status of knowledge. Cancer Res.

[3] Tao Q, Chan AT (2007). Nasopharyngeal carcinoma: molecular pathogenesis and therapeutic developments. Expert Rev Mol Med.

[4] Lo KW, Huang DP (2002). Genetic and epigenetic changes in nasopharyngeal carcinoma. Semin Cancer Biol.

[5] Bartel DP (2004). MicroRNAs: genomics, biogenesis, mechanism, and function. Cell.

[6] Bushati N, Cohen SM (2007). microRNA functions. Annu Rev Cell Dev Biol.

[7] Liu X, Lv XB, Wang XP, Sang Y, Xu S, Hu K (2012). MiR-138 suppressed nasopharyngeal carcinoma growth and tumorigenesis by targeting the CCND1 oncogene. Cell Cycle.

[8] Wang LJ, Chou YF, Chen PR, Su B, Hsu YC, Chang CH (2014). Differential miRNA expression in repeated recurrence of nasopharyngeal carcinoma. Cancer Lett.

[9] Liu N, Chen NY, Cui RX, Li WF, Li Y, Wei RR (2012). Prognostic value of a microRNA signature in nasopharyngeal carcinoma: a microRNA expression analysis. Lancet Oncol.

[10] Li T, Chen JX, Fu XP, Yang S, Zhang Z, Chen K (2011). microRNA expression profiling of nasopharyngeal carcinoma. Oncol Rep.

[11] Sengupta S, den Boon JA, Chen IH, Newton MA, Stanhope SA, Cheng YJ (2008). MicroRNA 29c is down-regulated in nasopharyngeal carcinomas, up-regulating mRNAs encoding extracellular matrix proteins. Proc Natl Acad Sci U S A.

[12] Luo Z, Zhang L, Li Z, Li X, Li G, Yu H (2012). An in silico analysis of dynamic changes in microRNA expression profiles in stepwise development of nasopharyngeal carcinoma. BMC Med Genomics.

[13] Pritchard CC, Cheng HH, Tewari M (2012). MicroRNA profiling: approaches and considerations. Nat Rev Genet.

[14] Plieskatt JL, Rinaldi G, Feng Y, Levine PH, Easley S, Martinez E, et al. Methods and matrices: approaches to identifying miRNAs for nasopharyngeal carcinoma. J Transl Med. 2014;12:3. doi:10.1186/1479-5876-12-3. 10.1186/1479-5876-12-3 PMC3895762 24393330

[15] Chang YY, Kuo WH, Hung JH, Lee CY, Lee YH, Chang YC, et al. Deregulated microRNAs in triple-negative breast cancer revealed by deep sequencing. Mol Cancer. 2015;14:36. doi:10.1186/s12943-015-0301-9. 10.1186/s12943-015-0301-9 PMC4351690 25888956

[16] Fend F, Raffeld M (2000). Laser capture microdissection in pathology. J Clin Pathol.

[17] Bonner RF, Emmert-Buck M, Cole K, Pohida T, Chuaqui R, Goldstein S (1997). Laser capture microdissection: molecular analysis of tissue. Science.

[18] Emmert-Buck MR, Bonner RF, Smith PD, Chuaqui RF, Zhuang Z, Goldstein SR (1996). Laser capture microdissection. Science.

[19] Wang S, Wang L, Zhu T, Gao X, Li J, Wu Y (2010). Improvement of tissue preparation for laser capture microdissection: application for cell type-specific miRNA expression profiling in colorectal tumors. BMC Genomics.

[20] Benjamini YHY (1995). Controlling the false discovery rate: a practical and powerful approach to multiple testing. J Roy Statist Soc Ser B.

[21] Livak KJ, Schmittgen TD (2001). Analysis of relative gene expression data using real-time quantitative PCR and the 2(−Delta Delta C(T)) Method. Methods.

[22] Kruger J, Rehmsmeier M (2006). RNAhybrid: microRNA target prediction easy, fast and flexible. Nucleic Acids Res.

[23] Lewis BP, Shih IH, Jones-Rhoades MW, Bartel DP, Burge CB (2003). Prediction of mammalian microRNA targets. Cell.

[24] Kertesz M, Iovino N, Unnerstall U, Gaul U, Segal E (2007). The role of site accessibility in microRNA target recognition. Nat Genet.

[25] John B, Enright AJ, Aravin A, Tuschl T, Sander C, Marks DS (2004). Human MicroRNA targets. Plos Biol.

[26] Kanehisa M, Goto S, Sato Y, Furumichi M, Tanabe M (2012). KEGG for integration and interpretation of large-scale molecular data sets. Nucleic Acids Res.

[27] Luscombe NM, Babu MM, Yu H, Snyder M, Teichmann SA, Gerstein M (2004). Genomic analysis of regulatory network dynamics reveals large topological changes[J]. Nature.

[28] Mao Q, Quan T, Luo B, Guo X, Liu L, Zheng Q (2015). MiR-375 targets KLF4 and impacts the proliferation of colorectal carcinoma. Tumour Biol.

[29] Kara M, Yumrutas O, Ozcan O, Celik OI, Bozgeyik E, Bozgeyik I (2015). Differential expressions of cancer-associated genes and their regulatory miRNAs in colorectal carcinoma. Gene.

[30] Wu Z, Wang H, Fang S, Xu C (2015). MiR-449c inhibits gastric carcinoma growth. Life Sci.

[31] Bai J, Zhu X, Ma J, Wang W (2015). miR-205 regulates A549 cells proliferation by targeting PTEN. Int J Clin Exp Pathol.

[32] Zhou C, Shen L, Mao L, Wang B, Li Y, Yu H (2015). miR-92a is upregulated in cervical cancer and promotes cell proliferation and invasion by targeting FBXW7. Biochem Biophys Res Commun.

[33] Peng H, Wang X, Zhang P, Sun T, Ren X, Xia Z (2015). miR-27a promotes cell proliferation and metastasis in renal cell carcinoma. Int J Clin Exp Pathol.

[34] Zhong Q, Wang T, Lu P, Zhang R, Zou J, Yuan S (2014). miR-193b promotes cell proliferation by targeting Smad3 in human glioma. J Neurosci Res.

[35] Hui AB, Bruce JP, Alajez NM, Shi W, Yue S, Perez-Ordonez B (2011). Significance of dysregulated metadherin and microRNA-375 in head and neck cancer. Clin Cancer Res.

[36] Qu C, Liang Z, Huang J, Zhao R, Su C, Wang S (2012). MiR-205 determines the radioresistance of human nasopharyngeal carcinoma by directly targeting PTEN. Cell Cycle.

[37] Li YQ, Ren XY, He QM, Xu YF, Tang XR, Sun Y (2015). MiR-34c suppresses tumor growth and metastasis in nasopharyngeal carcinoma by targeting MET. Cell Death Dis.

[38] Heinemann A, Zhao F, Pechlivanis S, Eberle J, Steinle A, Diederichs S (2012). Tumor suppressive microRNAs miR-34a/c control cancer cell expression of ULBP2, a stress-induced ligand of the natural killer cell receptor NKG2D. Cancer Res.

[39] Childs RW, Carlsten M (2015). Therapeutic approaches to enhance natural killer cell cytotoxicity against cancer: the force awakens. Nat Rev Drug Discov.

[40] Chen JJLS (2015). Has-miR-125a and 125b are induced by treatment with cisplatin in nasopharyngeal carcinoma and inhibit apoptosis in a p53-dependent manner by targeting p53 mRNA. Mol Med Rep.

[41] Li L, Wu J, Sima X, Bai P, Deng W, Deng X (2013). Interactions of miR-34b/c and TP-53 polymorphisms on the risk of nasopharyngeal carcinoma. Tumour Biol.

[42] Li Z, Chen X, Li L, Liu S, Yang L, Ma X (2012). EBV encoded miR-BHRF1-1 potentiates viral lytic replication by downregulating host p53 in nasopharyngeal carcinoma. Int J Biochem Cell Biol.

[43] Fu SM, Xu MX, Lin SM, Liang Z, Cai JH (2014). Association of cyclin D1 and survivin expression with sensitivity to radiotherapy in patients with nasopharyngeal carcinoma. Genet Mol Res.

[44] Xu Y, Shi Y, Yuan Q, Liu X, Yan B, Chen L (2013). Epstein-Barr Virus encoded LMP1 regulates cyclin D1 promoter activity by nuclear EGFR and STAT3 in CNE1 cells. J Exp Clin Cancer Res.

[45] Zhang LY, Ho-Fun LV, Wong AM, Kwong DL, Zhu YH, Dong SS (2013). MicroRNA-144 promotes cell proliferation, migration and invasion in nasopharyngeal carcinoma through repression of PTEN. Carcinogenesis.

[46] Lu J, He ML, Wang L, Chen Y, Liu X, Dong Q (2011). MiR-26a inhibits cell growth and tumorigenesis of nasopharyngeal carcinoma through repression of EZH2. Cancer Res.

[47] Fendri A, Kontos CK, Khabir A, Mokdad-Gargouri R, Ardavanis A, Scorilas A (2010). Quantitative analysis of BCL2 mRNA expression in nasopharyngeal carcinoma: an unfavorable and independent prognostic factor. Tumour Biol.

[48] Li Y, Yan L, Zhang W, Wang H, Chen W, Hu N (2014). miR-21 inhibitor suppresses proliferation and migration of nasopharyngeal carcinoma cells through down-regulation of BCL2 expression. Int J Clin Exp Pathol.

[49] Zhen Y, Liu Z, Yang H, Yu X, Wu Q, Hua S (2013). Tumor suppressor PDCD4 modulates miR-184-mediated direct suppression of C-MYC and BCL2 blocking cell growth and survival in nasopharyngeal carcinoma. Cell Death Dis.

[50] Xiang Q, Zhen Z, Deng DY, Wang J, Chen Y, Li J, et al. Tivantinib induces G2/M arrest and apoptosis by disrupting tubulin polymerization in hepatocellular carcinoma. J Exp Clin Cancer Res. 2015;34:118. doi:10.1186/s13046-015-0238-2. 10.1186/s13046-015-0238-2 PMC4603939 26458953

[51] Liu J, Xue H, Zhang J, Suo T, Xiang Y, Zhang W, et al. MicroRNA-144 inhibits the metastasis of gastric cancer by targeting MET expression. J Exp Clin Cancer Res. 2015;34:35. doi:10.1186/s13046-015-0154-5. 10.1186/s13046-015-0154-5 PMC4417226 25927670

[52] Lee BS, Kang S, Kim KA, Song YJ, Cheong KH, Cha HY (2014). Met degradation by SAIT301, a Met monoclonal antibody, reduces the invasion and migration of nasopharyngeal cancer cells via inhibition of EGR-1 expression. Cell Death Dis.

[53] Zhou HY, Wan KF, Ip CK, Wong CK, Mak NK, Lo KW (2008). Hepatocyte growth factor enhances proteolysis and invasiveness of human nasopharyngeal cancer cells through activation of PI3K and JNK. Febs Lett.

[54] Liu T, Li Q, Sun Q, Zhang Y, Yang H, Wang R (2014). MET inhibitor PHA-665752 suppresses the hepatocyte growth factor-induced cell proliferation and radioresistance in nasopharyngeal carcinoma cells. Biochem Biophys Res Commun.

[55] Li Y, Li W, He Q, Xu Y, Ren X, Tang X (2015). Prognostic value of MET protein overexpression and gene amplification in locoregionally advanced nasopharyngeal carcinoma. Oncotarget.

