# Correction to: Integrated analysis of microRNA regulatory network in nasopharyngeal carcinoma with deep sequencing

**DOI:** 10.1186/s13046-022-02311-7

**Published:** 2022-03-08

**Authors:** Fan Wang, Juan Lu, Xiaohong Peng, Jie Wang, Xiong Liu, Xiaomei Chen, Yiqi Jiang, Xiangping Li, Bao Zhang

**Affiliations:** 1 Department of Otolaryngology, Head and Neck Surgery, Nanfang Hospital, Southern Medical University, Guangzhou, 510515 China; 2 School of Public Health and Tropical Medicine, Southern Medical University, Guangzhou, 510515 China; 3 Department of Guangdong No.2 District, BGI Genomics Co.,Ltd, Shenzhen, 518083 China

**Correction to: J Exp Clin Cancer Res 35, 17 (2016)**

**https://doi.org/10.1186/s13046-016-0292-4**

Following publication of the original article [1], the authors identified a minor error in Fig. 4; specifically:

- Fig. 4b: The authors discovered that they had copied the results of different exposure times of the actin corresponding to CCDN1 to the actin assay corresponding to the CDK6 protein. The correct blot is now used to show the actin assay corresponding to the CDK6 protein.

The corrected figure is given here. The correction does not have any effect on the final conclusions of the paper.

**Fig. 4:**
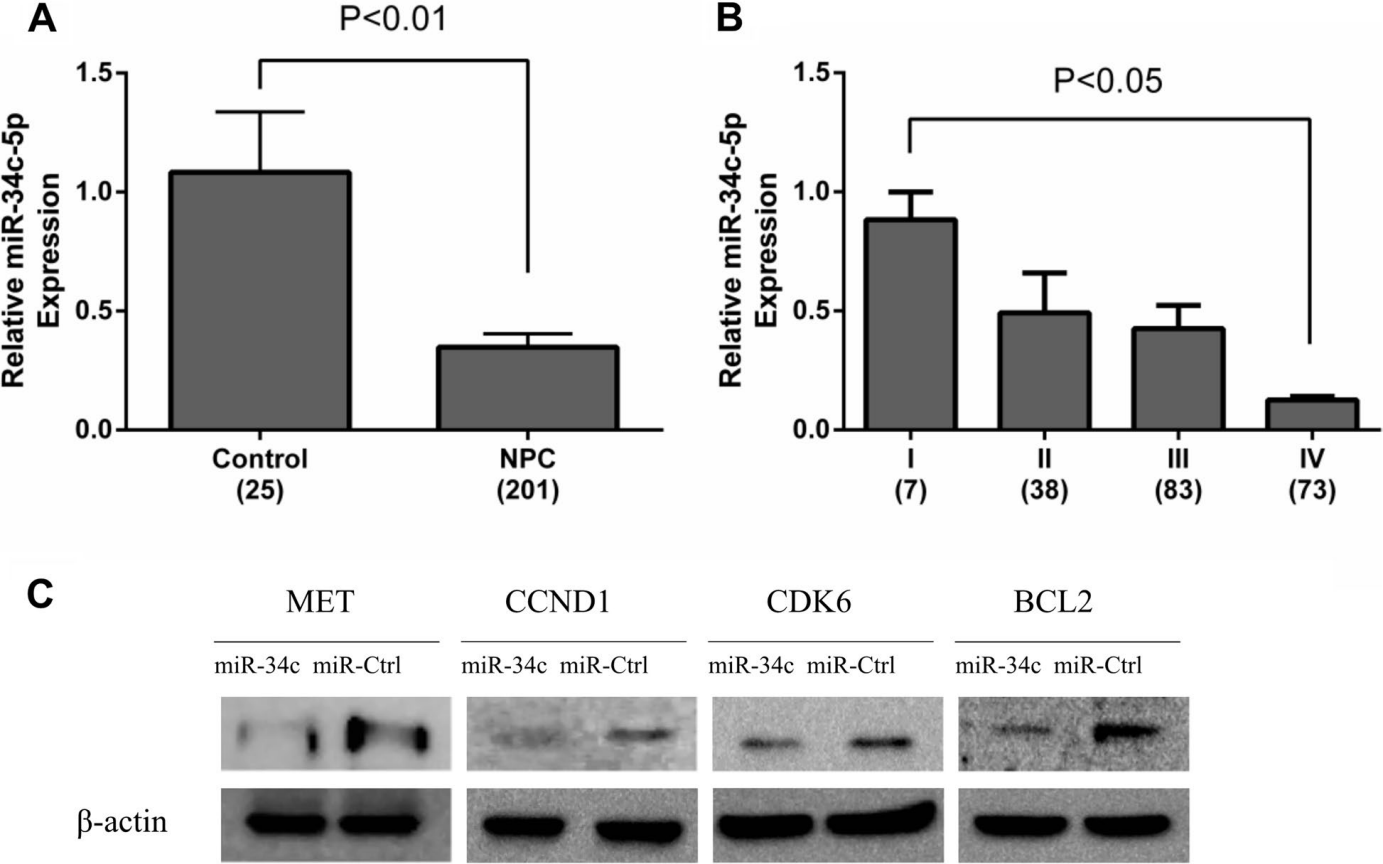
MiR-34c levels during NPC progression and target genes validation. **a** The expression level of miR-34c-5p in human NPC specimens compared with control biopsy samples. **b** miR-34c-5p expression was higher in stage I, whereas stages II-IV had lower levels. **c** The protein expression levels of MET, CCND1, CDK6 and BCL2 in miR-34c mimic transfected CNE-2 cells were lower than the controls. Western blot was independently repeated at least three times. MicroRNA abundance was normalised to U6 RNA. Statistical analysis was performed using the t-tests (**a, c**) and the one-way ANOVA (**b**)
