# Supplementary material for: Integrated analysis of microRNA regulatory network in nasopharyngeal carcinoma with deep sequencing
Source: J Exp Clin Cancer Res. 2016 Jan 22;35:17. doi: 10.1186/s13046-016-0292-4 (PMC4722718; doi:10.1186/s13046-016-0292-4)
Supplement: Additional file 2 — Figure S1. MiR-34c-5p have binding sites on the 3’-UTR of target genes. A. Complementary binding sites of miR-34c-5p on the 3’-UTR of CCND1. B. Complementary binding sites of miR-34c-5p on the 3’-UTR of BCL2. C. Complementary binding sites of miR-34c-5p on the 3’-UTR of CDK6. D. Complementary binding sites of miR-34c-5p on the 3’-UTR of MET. The vertical lines indicate the complementary base pairs (which have also been highlighted). (DOC 5879 kb) [file 13046_2016_292_MOESM2_ESM.doc]

**
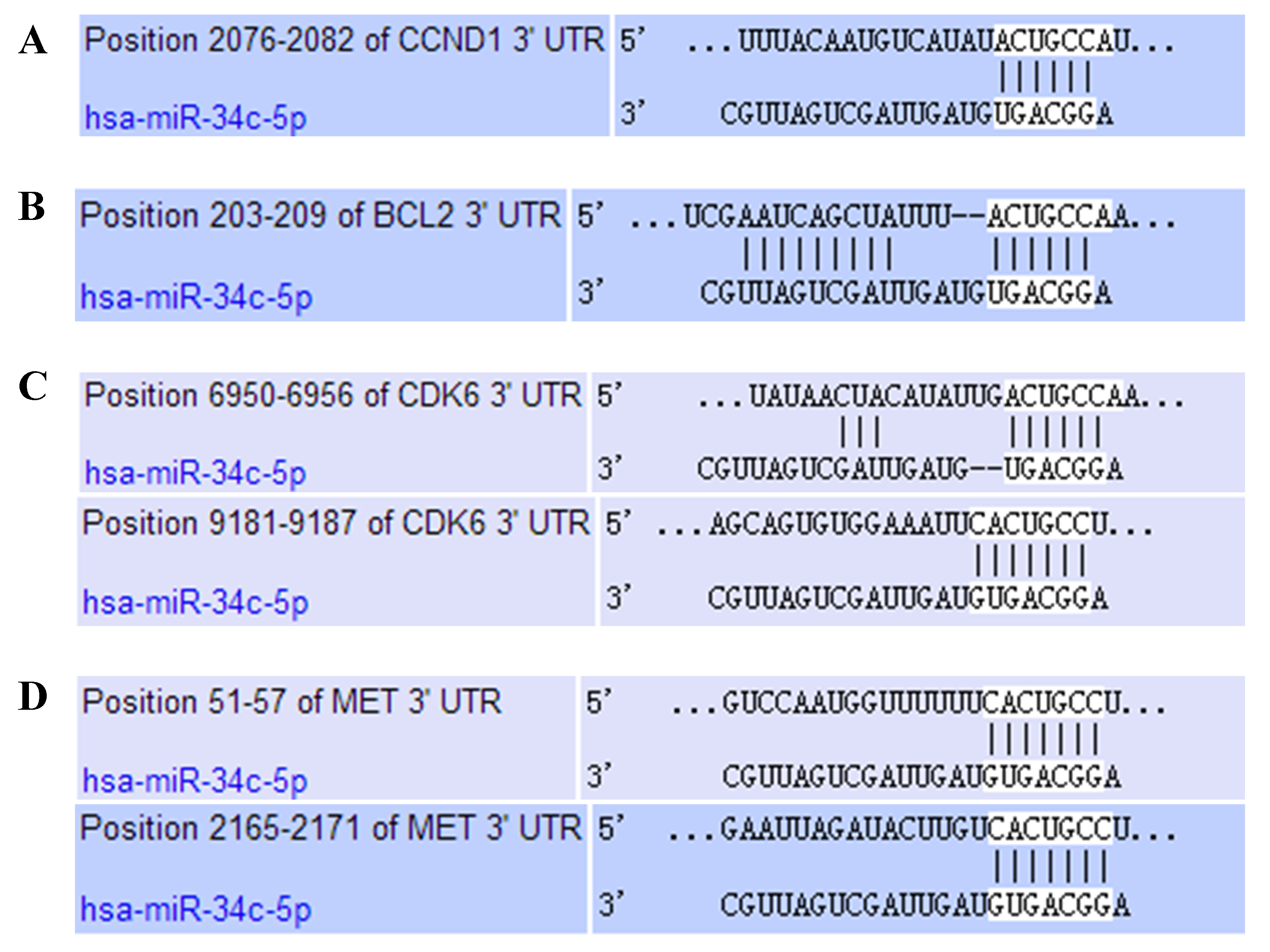
**

**Additional file 2: Figure S1. MiR-34c-5p have binding sites on the 3’-UTR of target genes. A.** Complementary binding sites of miR-34c-5p on the 3’-UTR of CCND1. **B.** Complementary binding sites of miR-34c-5p on the 3’-UTR of BCL2. **C.** Complementary binding sites of miR-34c-5p on the 3’-UTR of CDK6. **D.** Complementary binding sites of miR-34c-5p on the 3’-UTR of MET. The vertical lines indicate the complementary base pairs (which have also been highlighted).
